# Design of Viologen-Based Liquid Crystals Exhibiting Bicontinuous Cubic Phases and Their Redox-Active Behavior

**DOI:** 10.3390/ma10111243

**Published:** 2017-10-27

**Authors:** Tsubasa Kobayashi, Takahiro Ichikawa

**Affiliations:** 1 Department of Biotechnology, Tokyo University of Agriculture and Technology Nakacho, Koganei, Tokyo 184-8588, Japan; s178690r@st.go.tuat.ac.jp; 2 Precursory Research for Embryonic Science and Technology (PRESTO), Japan Science and Technology Agency (JST) 4-1-8 Honcho, Kawaguchi 332-0012, Japan

**Keywords:** viologen, liquid crystal, bicontinuous cubic phase, gyroid, zwitterion, photo patterning

## Abstract

We have succeeded in developing viologen-based liquid-crystalline materials forming bicontinuous cubic phases. They are composed of amphiphilic zwitterions with a viologen ionic-head-group and sulfonyl-imide-type acids. In the bicontinuous cubic liquid-crystalline assemblies, the ionic-head groups of the amphiphiles align along a gyroid minimal surface, forming a 3D continuous viologen layer. The ionic state of the viologen-moieties can be tuned from a dication state (V^2+^) to a radical mono cation state (V^1+•^) by UV irradiation and/or electric field. This redox reaction proceeds in bulk, accompanying the change of their color from colorless to purple. Interestingly, they preserve the 3D molecular assembled structures beyond the redox reaction, which has been confirmed by polarizing optical microscopy and X-ray diffraction measurements.

## 1. Introduction

Viologen is a class of ionic compounds with bipyridinium dication structures (V^2+^). It is a representative redox-active molecule showing specific chemical response to photo- and electro- stimuli. For example, it turns into a stable radical mono cation state (V^1+•^) by UV irradiation, accompanying a color change from colorless to purple. The V^1+•^ state is quenched by reverse reactions or certain chemicals (a representative material is oxygen), turning back into the initial state (V^2+^). Since the reduction and oxidation reaction can be induced repeatedly by each stimulus, viologen is known as a stable redox-active group and used as a building block for various functional materials, such as electrochromic [1,2,3,4,5] and photoelectric conversion materials [6,7].

Recently, there has been increasing attention paid to the ordering of viologen groups because ordered viologen groups exhibit specific functions and enhanced properties [8]. One of the methods used to align viologen groups is to introduce them into the side chain of polymers [6,7]. Murray reported that the successive alignment of viologen groups along a polymer backbone enables a fast, self-electron transfer reaction. These materials are applicable for redox-flow batteries [9]. Another useful strategy for aligning viologen groups into an ordered manner is to endow them with liquid crystallinity through suitable molecular design [10,11,12,13,14,15,16,17]. Since liquid-crystalline (LC) materials have the ability to spontaneously form molecular ordered structures, they have established themselves as a significant class of nanostructured soft materials. Representative molecular designs of LC viologen molecules are summarized in Figure S1. For example, Kijima and Shirakawa developed viologen derivative LC molecules with a pyrole group at the terminal of the molecules [10]. The polymerization of the pyrole moiety yields LC polymers forming smectic (Sm) phases, which act as both 2D ions and electron transporters. As another example, Beneduci and co-workers designed ionic liquid crystals based on thienoviologens [11,15]. These liquid crystals exhibit Sm and columnar (Col) phases depending on the alkyl chain lengths. Fast and reversible bulk electrofluorochromic responses have been observed for both in the Sm and Col phases.

In the last decade of study, we have focused on the functionalization of bicontinuous cubic (Cub_bi_) liquid crystals because they form well-ordered nanostructures with cubic periodicity and 3D continuity, and then exhibit structure-dependent properties and functions [18,19,20,21,22]. In an aim to convert these structural features to functional advantages, we developed some new molecular design principles [23,24,25,26,27]. Among several principles, the use of zwitterions as a building block of LC molecules is a powerful strategy for creating Cub_bi_ LC materials with high probability. Since zwitterions have the ability to form homogeneous complexes with various type of acids, it is possible to tune the self-organization behavior of zwitterionic LC molecules by the addition of suitable acids. Amphiphilic zwitterions designed by our group are shown in Figure S2. These zwitterions form Cub_bi_ phases in the presence of some selected acids or lithium salts. In particular, pyridinium-based zwitterions (**PyZI**) are representative molecules that exhibit thermotropic Cub_bi_ phases in the presence of sulfonyl-imide-type acids, such as bis(trifluoromethane sulfonyl)imide (**HTf_2_N**). 

**Figure 1:**
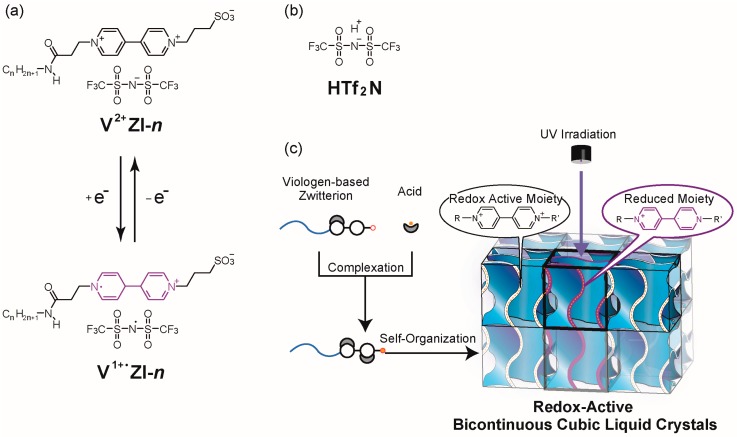
(**a**) Design of amphiphilic molecules with a viologen-based zwitterionic head group (**V^2+^ZI-*n***) and their radical mono cation state (**V^1+•^ZI-*n***); (**b**) Molecular structure of **HTf_2_N**; (**c**) Equimolar mixing of **V^2+^ZI-*n*** and **HTf_2_N** forming redox-active bicontinuous cubic liquid-crystalline phases.

Considering these molecular examples of amphiphilic zwitterions, in the present study, we newly designed amphiphilic zwitterions **V^2+^ZI-*n*** by substituting the pyridinium cation of **PyZI** to a viologen dication for the development of Cub_bi_ liquid crystals showing a redox-active property. The concept in the present study and the molecular design of **V^2+^ZI-*n*** are summarized in Figure 1a–c. The synthetic scheme of **V^2+^ZI-*n*** is shown in the Supplementary Materials. *n* indicates the number of carbon atoms in the alkyl chain. Below, we describe the control of the self-organization behavior of **V^2+^ZI-*n*** with the addition of acids. Moreover, their redox-active properties are discussed. 

## 2. Results and Discussion

Generally, block molecules consisting of ionic and non-ionic parts have the potential to self-organize into LC ordered states through nanosegregation of the incompatible parts [28,29]. The adjustment of the number and length of alkyl chains is an often-used approach for controlling the self-organization behavior of these molecules because the volume balance between the incompatible parts is a driving force governing the nanosegregated mesophase pattern [23,24,25,26,27]. To realize the design of viologen-based amphiphilic molecules exhibiting Cub_bi_ LC phases, we employed the control of two factors: one is the adjustment of the alkyl chain length and the other is the selection of acids. 

**Figure 2:**
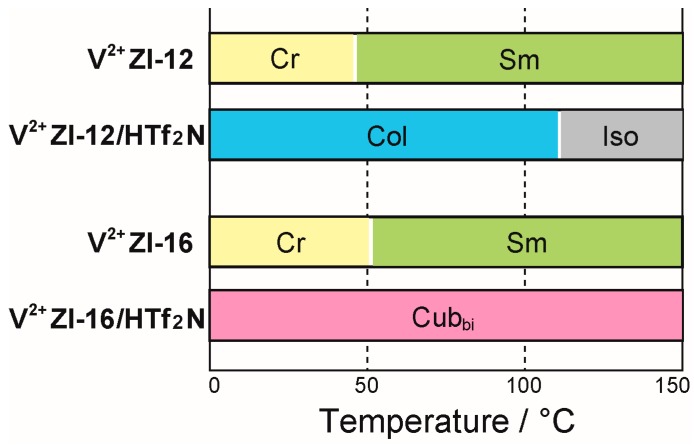
Thermotropic liquid-crystalline behaviors of **V^2+^ZI-*n*** and their mixtures with **HTf_2_N**. Cr, crystalline; Sm, smectic; Col, columnar; Cub_bi_, bicontinuous cubic; Iso, isotropic.

**V^2+^ZI-*n*** are obtained as white solids showing thermotropic Sm LC phases. In an effort to control their LC properties to form Cub_bi_ phases, we mixed them with several acids. The mixtures of **V^2+^ZI-*n*** and acids were successfully prepared by dissolving the two components into methanol with a small amount of water and subsequent evaporation of the methanol/water. The obtained mixtures showed thermotropic LC behaviors that greatly vary depending on the selection of the acid species. Here, we show the LC behaviors of **V^2+^ZI-*n***/acid mixtures when using **V^2+^ZI-*n*** and a class of sulfonyl-imide derivatives (**H-A**) as acids. There are definite differences in the thermotropic LC behaviors of **V^2+^ZI-*n*/H-A** mixtures depending on the added acid species (selection of **H-A**) and the length of the alkyl chain (adjustment of *n*) (Figure S5a,b in the Supplementary Materials). Themotropic LC behaviors of **V^2+^ZI-*n*** and their mixtures with **HTf_2_N** are summarized in Figure 2. We strongly point out that the tuning of these parameters leads to the exhibition of Cub_bi_ phases successfully. For example, an equimolar mixture of **V^2+^ZI-16** and **HTf_2_N** (**V^2+^ZI-16/HTf_2_N**) exhibits a Cub_bi_ phase from 0 to 150 °C, while that of **V^2+^ZI-12** and **HTf_2_N** (**V^2+^ZI-12/ HTf_2_N**) shows a Col phase from 0 to 110 °C. Although there have been several reports on the design of viologen-derived liquid crystals [9,10,11,12,13,14,15], to the best of our knowledge, this is the first example of those showing thermotropic Cub_bi_ phases. Considering a general recognition of the difficulty in designing thermotropic Cub_bi_ liquid crystals [23,24,25,26,27,28,29], it is considered that our LC molecular design principle based on amphiphilic zwitterions is a versatile strategy for endowing functional molecules with Cub_bi_ liquid crystallinity.

In general, viologen-derived compounds are known to turn their own color from colorless to purple when they are reduced by UV irradiation and/or electric field. This color change comes from reduction reactions from dication states (V^2+^) to radical mono cation states (V^1+•^). Our interest here is in the following two questions. One is whether these **V^2+^ZI-*n*/H-A** mixtures show reducibility in Cub_bi_ LC states by UV irradiation (λ = around 400–500 nm) and/or electric field. The other is whether the viologen-based Cub_bi_ liquid crystals preserve their 3D molecular assembled nanostructures after reduction. Focusing on these two points, we examined the material properties of the **V^2+^ZI-*n*/H-A** mixtures.

**Figure 3:**
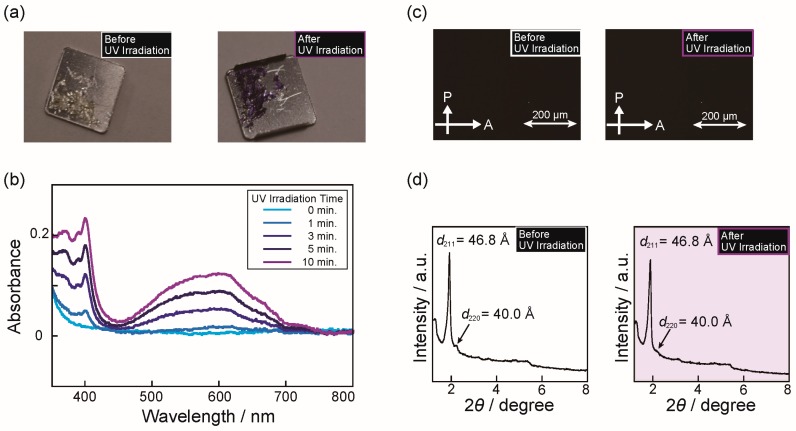
(**a**) Color change of **V^2+^ZI-16/HTf_2_N** mixture before and after UV irradiation for 15 min; (**b**) UV-vis spectra of **V^2+^ZI-12/HTf_2_N** mixture before and after UV irradiation; (**c**) Polarizing optical microscope images of **V^2+^ZI-16/HTf_2_N** mixture before and after UV irradiation; (**d**) X-ray diffraction patterns of **V^2+^ZI-16/HTf_2_N** mixture at 30 °C in the Cub_bi_ phase before and after UV irradiation.

The reduction behavior of **V^2+^ZI-*n*/H-A** mixtures upon UV irradiation can be clearly confirmed by the naked eye as a color change from colorless to purple (Figure 3a). To quantitatively evaluate the progress of the photo-reduction, we performed UV-vis spectroscopy measurement for **V^2+^ZI-12/HTf_2_N** mixtures in the LC states before and after the UV irradiation. The spectra changes of **V^2+^ZI-12/HTf_2_N** mixtures are shown in Figure 3b. Two absorption bands gradually appear at around 400 nm and 600 nm, which is indicative of the incremental formation of the V^1+•^ state [13]. Their redox behaviors against electric field has been also examined. For this examination, **V^2+^ZI-*n*/HTf_2_N** mixtures were sandwiched between an indium tin oxide (ITO) glass and an ITO glass coated with doped electron conductive polymers, and then a constant voltage was applied to the sample. Further details of the experimental methods and materials are described in the Supplementary Materials (Figure S12). An immediate color change, which is much faster than reduction by UV irradiation, was found with the experiment (Figure S13).

With the aim of examining whether **V^2+^ZI-*n*/H-A** mixtures preserve the Cub_bi_ LC nanostructures after reduction, we performed polarizing optical microscopy (POM) measurements before and after UV irradiation (Figure 3c). Before UV irradiation, **V^2+^ZI-16/HTf_2_N** mixture formed a Cub_bi_ phase (Cub_bi_ (V^2+^)) that was colorless and had no birefringence (black texture when observed with POM, Figure 3c, left). After UV irradiation, the preservation of black texture with no birefringence was confirmed by POM observation (Figure 3c, right). A color change from colorless to purple was observed by the naked eye. Figure 3d shows the XRD patterns of the **V^2+^ZI-16/HTf_2_N** mixture before and after UV irradiation. Little differences were found in the two XRD patterns. For example, the XRD pattern of the **V^2+^ZI-16/HTf_2_N** mixture in the Cub_bi_ (V^2+^) state shows two intense peaks at 2θ = 1.89 and 2.20 corresponding to (211) and (220) reflections of *Ia*3*d* (gyroid type) cubic symmetry, and a similar diffraction pattern was observed for the same sample in the Cub_bi_ (V^1+•^) state, while a weakening of the (220) reflection was found. Similar experiments have been performed for other mixtures forming Col phases, such as with the **V^2+^ZI-12/HTf_2_N** mixture. In addition to the **V^2+^ZI-16/HTf_2_N** mixture, the formation of Col structures and its preservation after photo-reduction were also confirmed by POM and XRD measurements (See the Supplementary Materials, Figure S11). These results indicate that the strong intermolecular interactions between **V^2+^ZI-*n*** molecules in the assembled states suppress the rearrangement of the component molecules, leading to the preservation of molecular assembled structures.

**Figure 4:**
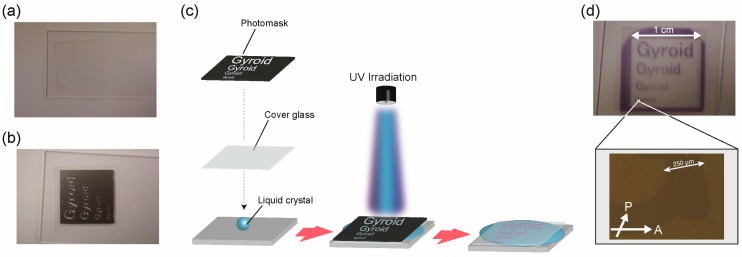
(**a**) Cub_bi_ (V^2+^) sample sandwiched between a glass plate and a cover glass; (**b**) A picture of the patterned photomask; (**c**) Experimental procedure for UV-pattering experiment; (**d**) A Cub_bi_ (V^2+^)/Cub_bi_ (V^1+•^) sample patterned by UV irradiation.

To examine whether or not it is possible to perform UV-reduction only in the local area, we carried out a UV irradiation experiment through a patterned mask. The photomask employed was carved on several sizes of “Gyroid”. The minimum width of the letter “G” was less than 1 mm. Gyroid is one of the name of 3D periodical minimal surfaces. In the Cub_bi_ LC assembly formed by **V^2+^ZI-*n*/H-A** mixtures, viologen groups were successively aligned along with a gyroid minimal surface. The Cub_bi_ (V^2+^) sample was sandwiched between a slide glass and a cover glass (Figure 4a). The photomask was placed on the cover glass (Figure 4b). The sandwiched samples were irradiated by a UV lamp with high intensity for 15 min. A schematic illustration of the experimental method is shown in Figure 4c. The photo-patterned sample was successfully obtained, in which all the sizes of purple colored “Gyroid” domains are seen contrasting with colorless domains (Figure 4d). Since the macroscopic diffusion coefficients in the Cub_bi_ LC sample are significantly suppressed by the strong intermolecular interactions in the assembled states, the photo-patterned states are stable with high resolution for a long period of time, although the patterned letters disappear through reverse electron-transfer reactions after several hours.

Based on the above results, we envision that if a certain physicochemical property of the viologen derivative liquid crystals can be largely changed by reduction, this change can be exploited to induce switchable functions. As a potential physicochemical property, we focused on the hydrophilicity of the viologen groups. It is known that the hydrophilicity of viologen decreases as it is reduced from V^2+^ to V^1+•^ states [30].

**Figure 5:**
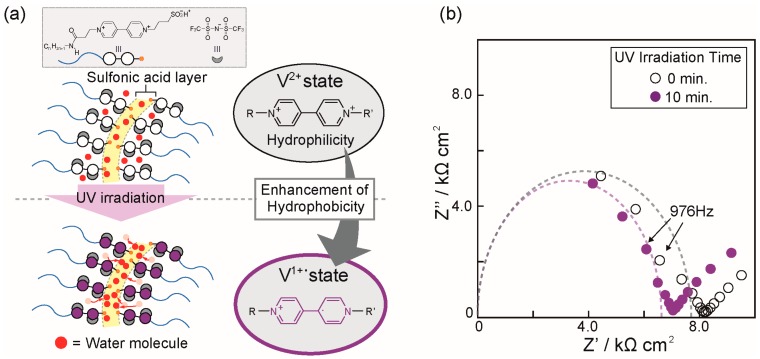
(**a**) An illustration of a concept for photo-responsive switchable proton conduction materials; (**b**) Cole-cole plots of the **V^2+^ZI-12/HTf_2_N** mixture before and after 10 min of UV irradiation.

In the previous study, we reported that our material design, which is the combination of amphiphilic zwitterions and acids, provides a unique gyroid minimal surface with a great potential to function as a proton conduction pathway because the mixtures of amphiphilic zwitterions and acids provide a specific situation where the hydrophilic sulfonic acid groups of the amphiphilic zwitterions locate regularly on the 3D continuous gyroid minimal surface. To endow this surface with effective proton conduction ability, the incorporation of a small amount of water into the materials and the alignment of the water molecules exclusively in the hydrophilic sulfonic acid layer are essential, as cooperative hydrogen networks between the sulfonic acid groups and water molecules are effective for inducing proton hopping conduction [24,26]. For the realization of this concept, it is expected to be significant to construct a hydrophobic situation on both sides of the hydrophilic layer in order to shed water molecules and concentrate them into the hydrophilic layer. Considering this background of the amphiphilic zwitterions/acid systems, we expect that the hydrophobicity change could be used to control the position of water molecules in the sulfonic acid layer and subsequently switch the proton conductivity. Schematic illustration of this concept is shown in Figure 5a. Our hypothesis is that the enhancement of hydrophobicity upon the change from the V^2+^ to V^1+•^ states may result in the enhancement of the water shedding effect of the viologen layers, which leads to an increase of the number of water molecules along the sulfonic acid layer. In order to test this, we performed ion conduction measurements by controlling UV irradiation. The sample for ion conduction measurements was prepared with a slightly-hydrous state using the hygroscopic nature of **V^2+^ZI-*n*/HTf_2_N** mixtures. Since it was difficult to prepare hydrated samples forming Cub_bi_ phases, we used a hydrated sample of the **V^2+^ZI-12/HTf_2_N** mixture that forms a Col phase. For the verification of our hypothesis, it was required to keep the water content in the sample during the measurements because the slight increase of the water content resulted in a large increase of the ionic conductivity in the present system. Therefore, a hydrated **V^2+^ZI-12/HTf_2_N** sample was prepared by keeping it under a controlled condition, with a temperature of 28 °C and a relative humidity of 75%, until reaching an equilibrium state before the measurements. AC impedance analysis was performed for the sample in the equilibrium state before and after UV irradiation. Cole-cole plots for the obtained results before and after UV irradiation are shown in Figure 5b. These plots exhibit approximate semicircles. It can be seen that the semicircle becomes smaller upon UV irradiation, which means that the resistance in the material decreases. This result suggests that ions can move slightly more smoothly in the V^1+•^ state than in the V^2+^ state, which supports our hypothesis but is far from conclusive. For definitive confirmation, further experiments will be required. We believe that optimization of the molecular design in the amphiphilic zwitterions/acid systems will lead to the development of ion conductive materials showing more drastic switching behavior. 

In this study, we applied our material design to use a combination of amphiphilic zwitterions and acids to align viologen groups in a 3D-ordered and continuous manner. We believe that this material design is versatile, not only for viologen groups but also for a variety of functional groups with unique functions, such as photo-, electro-, magnetic-, and conductive-properties. 

## 3. Materials and Methods

### 3.1. Preparation of **V^2+^ZI-n/H-A** Mixtures

A synthetic scheme for **V^2+^ZI-*n*** is shown in the Supplementary Materials. Various acids were synthesized according to the literature [26]. Mixtures of the **V^2+^ZI-*n*** and acids were successfully prepared by dissolving the two components into methanol with a small amount of water and subsequent evaporation of the methanol/water. The water contents of the obtained mixtures were evaluated to be around 2–3 wt % by Karl Fischer titration.

### 3.2. Thermotropic Liquid-Crystalline Properties

Thermotropic liquid-crystalline behaviors of the mixtures were examined in the temperature range from room temperature to 150 °C by POM and XRD measurements. Some obtained results are shown in the Supplementary Materials. Differential scanning calorimetry measurements were also performed for the mixtures. 

### 3.3. UV Irradiation

As a UV source, a xenon lamp with specific wavelength cut filter was used. For the preparation of the samples for XRD and POM measurements, UV irradiation with strong power was used to rapidly induce the reduction reaction. On the other hand, for the preparation of the samples for the UV-vis spectroscopy measurements and the ion conduction measurements, UV irradiation with weak power was used in order to reduce the heat generation.

## 4. Conclusions

We have succeeded in the molecular design of liquid-crystalline (LC) viologen derivatives forming bicontinuous cubic (Cub_bi_) phases. These molecules are composed of amphiphilic zwitterions with viologen cation and sulfonyl-imide-type acids. In the Cub_bi_ LC assemblies, their viologen dication (V^2+^) moieties, aligning along with a gyroid minimal surface, form a continuous 3D ionic layer. These liquid crystals show redox-active properties responding to UV irradiation and/or electric field, leading to the reduction of V^2+^ moieties to monocation (V^1+•^) states. It is noteworthy that the reduction reaction proceeds, maintaining the preformed Cub_bi_ LC nanostructures, while the change of color from colorless to purple is observed. The photo-reduction can be induced in the limited local area by using patterned photomasks. The present material design has great potential for the development of novel nanostructured materials with 3D periodicity that are applicable for information storage and optical materials.

## Supplementary Materials

The following are available online at www.mdpi.com/1996-1944/10/11/1243/s1, Figure S1: Molecular structures of viologen-based thermotropic liquid crystals [S1-1-S1-5], Figure S2: Molecular structures of zwitterionic liquid crystals designed by our group, Scheme S1: Synthetic scheme for **V2+ZI-n**, Figure S3: 1H-NMR spectrum of **V2+ZI-12**. An equimolar amount of LiTf2N is also dissolved in order to increase the solubility of **V2+ZI-12**, Figure S4: 1H-NMR spectrum of **V2+ZI-16**. An equimolar amount of LiTf2N is also dissolved in order to increase the solubility of **V2+ZI-16**, Figure S5: POM images of the mixtures of **V2+ZI-16** and various acids; (a) imide-type acids with a long alkyl chain. The mixture shows a Sm phase; (b) imide-type acids with an aromatic ring. The mixture shows a Cubbi phase from 100 to 150 °C and a Col phase from room temperature to 100 °C, Figure S6: POM images of hydrated **V2+ZI-16/HTf2N** mixtures (water contents are 5–10 wt %); (a) at 120 °C in the Cubbi phases; (b) at 95 °C in the Cubbi phases; (c) at 90 °C in the Cubbi and Col phases; (d) at 60 °C in the Col phases, Figure S7: POM images of **V2+ZI-12** (left) and **V2+ZI-16** (right) showing Sm phases at 40 °C, Figure S8: POM images of **V2+ZI-12/HTf2N**; (a) at 115 °C in the Isotropic state; (b) at 110 °C starting in the Col phase; (c) at 80 °C in the Col phases; (d) at 60 °C in the Col phase, Figure S9: DSC thermograms of; **V2+ZI-12** (left) and **V2+ZI-16** (right) on heating and cooling, Figure S10: DSC thermograms of; **V2+ZI-12/HTf2N** (left); **V2+ZI-16/HTf2N** (right) on heating and cooling, Figure S11: (a) POM pictures before and after UV irradiation. Birefringence formed by each Col domain was maintained while their color turn to purple from colorless. (b) XRD patterns before and after UV irradiation. Two diffraction patterns attributed to (100), (200) of Col plane are observed, Figure S12: Schematic illustration for voltage application experiments. As applying to voltage, color change immediately occurred to purple from colorless, Figure S13: (a) A picture of indium thin oxide glass coated conductive polymer (PEDOT-PSS) electrode. (b) The sample was sandwiched by an indium thin oxide glass and a prepared glass before applying voltage at 140 °C. (c) Color changed sample after applying voltage at 30 °C, Figure S14: Ionic conductivity of **V2+ZI-12/HTf2N** mixture. UV was irradiated for 5 to 20 min. We checked the color change from colorless to purple accompanying with UV irradiation.
