# Supplementary material for: Design of Viologen-Based Liquid Crystals Exhibiting Bicontinuous Cubic Phases and Their Redox-Active Behavior
Source: Materials (Basel). 2017 Oct 27;10(11):1243. doi: 10.3390/ma10111243 (PMC5706190; doi:10.3390/ma10111243)
Supplement: Supplementary file 1 [file materials-10-01243-s001.pdf]

Supplementary Materials: Design of Viologen-Based Liquid Crystals Exhibiting Bicontinuous Cubic Phases and Their Redox-Active Behavior

Tsubasa Kobayashi, Takahiro Ichikawa

Abbreviated words

LC	Liquid-crystallline
Cr	Crystal
Sm	Smectic
Col	Columnar
Cub_{bi}	Bicontinuous cubic
POM	Polarizing optical microscope
XRD	X-ray diffraction
DSC	Differential scanning calorimetry

1. Representative viologen-based liquid-crystalline molecules

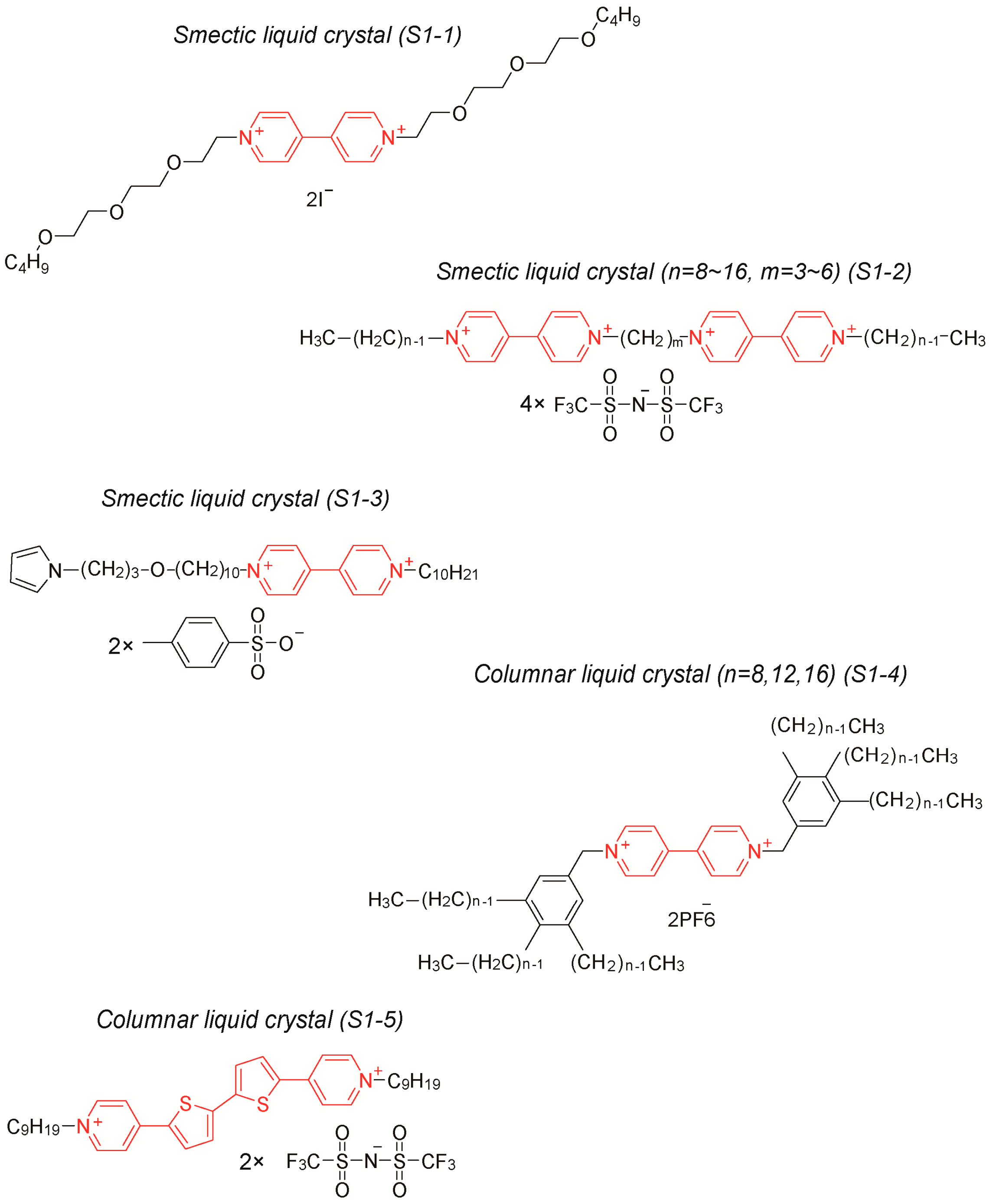

Figure S1. Molecular structures of viologen-based thermotropic liquid crystals [S1-1-S1-5].

2. Liquid-crystalline zwitterionic amphiphilic molecules designed by our group

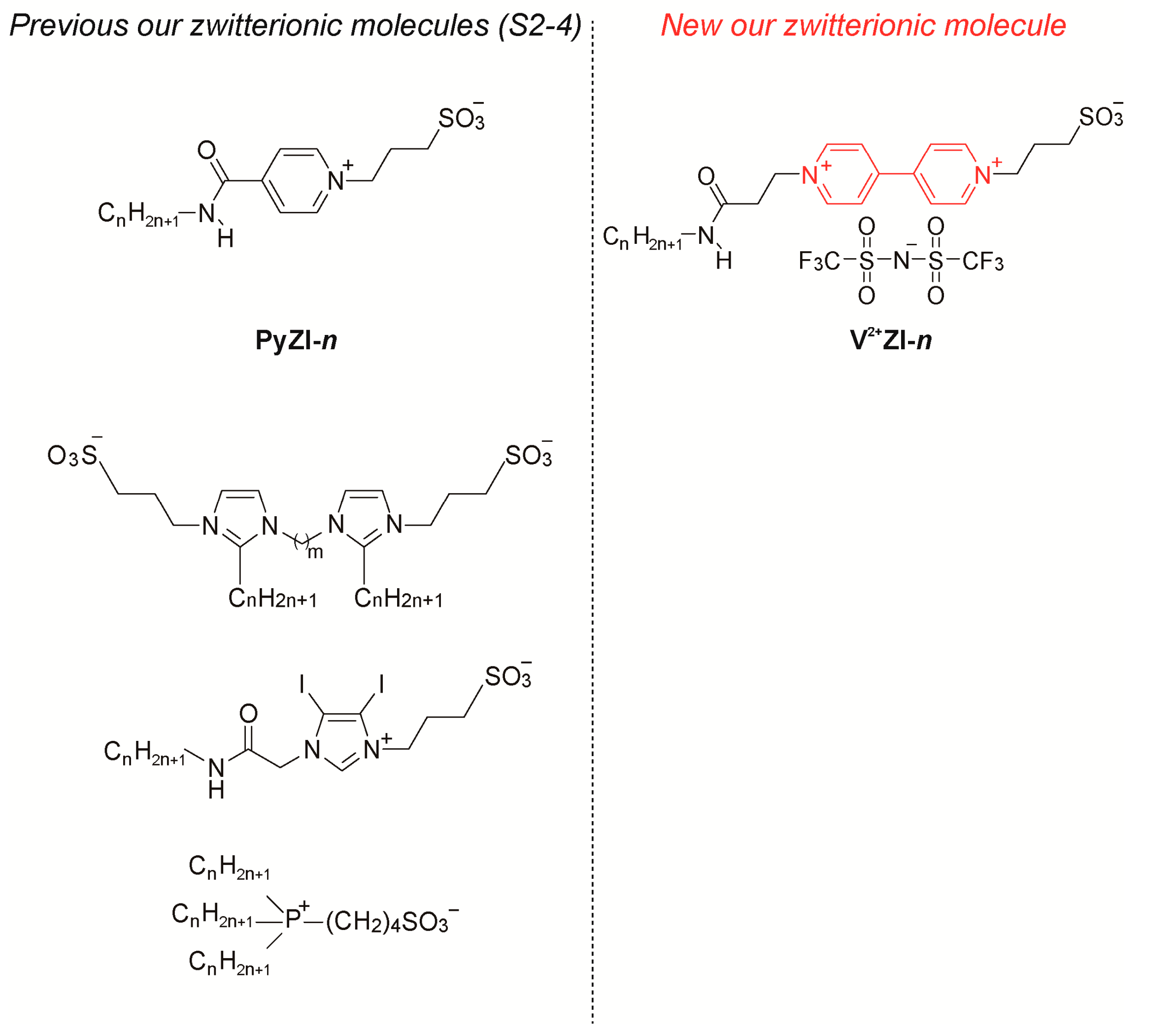

Figure S2. Molecular structures of zwitterionic liquid crystals designed by our group.

3. General Procedures

^{1}H NMR spectra were obtained on a JNM-ECA500 (JEOL). Thermal properties of the present materials were examined with DSC-6220 (Seiko Instruments) at a heating and cooling rate of 5 °C min^{-1}. The textures of liquid-crystalline materials were obtained with a polarizing optical microscope, Olympus BX51 equipped with a Lincam hot-stage. X-ray diffraction measurement was performed using a Rigaku Smart-Lab with CuKα radiation. UV was irradiated by Asahi Spectra by taking distance approximately 30 cm.

4. Materials

All chemical reagents and solvents were commercially obtained and used as received. All reactions were performed in anhydrous solvents under argon atmosphere.

5. Synthesis Scheme

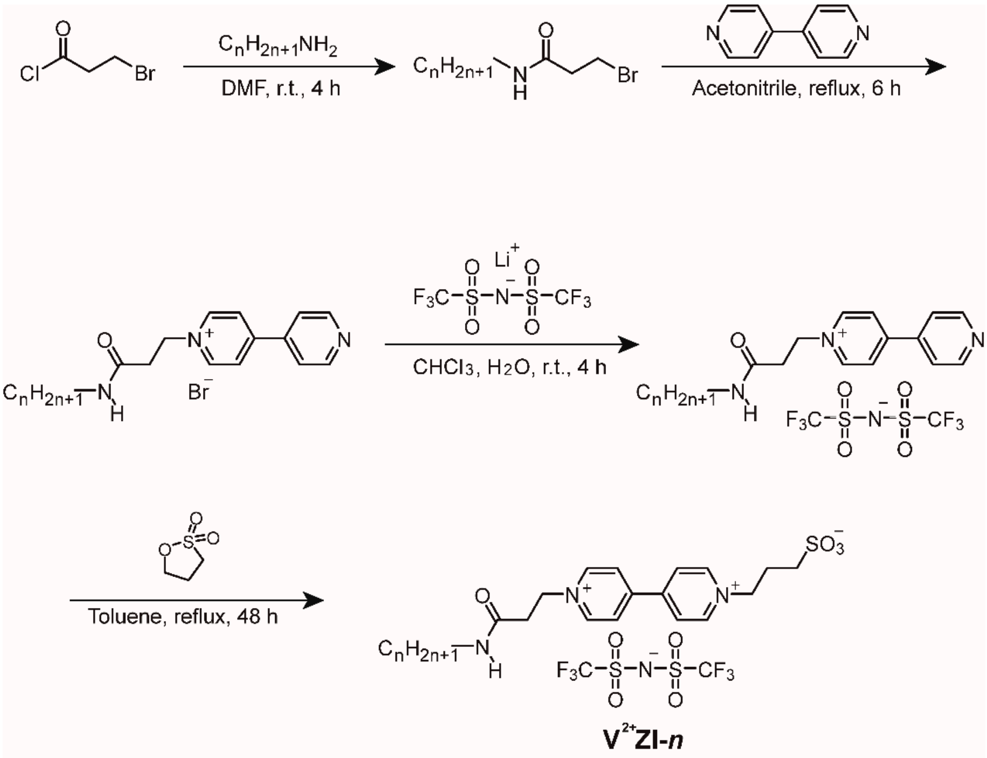

Scheme S1. Synthetic scheme for **V²⁺ZI-*n***.

3-Bromo-N-dodecyl propane amine

A solution of dodecyl amine (3.25 g; 18 mmol) and triethylamine (2.4 ml; 18 mmol) in *N*,*N*-dimethylformamide (20 ml) was added dropwise to the solution of 3-bromopropionyl chloride in *N*,*N*-dimethylformamide (20 ml) at 0 °C. The mixture was stirred for 4h at room temperature. Then water and ethyl acetate were added to the mixture, and organic phase was separated with water phase. After the resulting organic phase was dried over $MgSO_4$, the solution was evaporated. The crude product was purified be flash column chromatography (silica gel, eluent: chloroform/methanol = 19/1) to give 3-bromo-*N*-dodecyl propane amine (5.2 g; 16 mmol 89%) as a white solid.

^{1}H NMR (400 MHz, $CDCl_3$): δ = 5.62 (d, J = 10.0 Hz, 1H), 3.81 (t, J = 4.3 Hz, 1H), 3.28 (t, J = 6.8 Hz, 2H), 2.6 (t, J = 4.3 Hz, 1H), 1.51-1.25 (m, 22H), 0.88 (t, J = 9.2 Hz, 3H).

[1-(3-(Dodecylamino)-3-oxopropyl)-[4,4′-bipyridin]-1-ium] [bis((trifluoromethyl)sulfonyl)amide]

4,4'-Bipyridyl (3.8 g; 24 mmol) was added to the solution of 3-bromo-*N*-dodecyl propane amine (5.2 g; 16 mmol) in acetonitrile (20 ml). The mixture was stirred vigorously for 6h under reflux. Then the acetonitrile was removed by rotary evaporation, and crude product was stirred for 48h with excess amount of lithium bis((trifluoromethyl)sulfonyl)imide ($LiTf_2N$) in chloroform and water solution. The organic phase was separated and dried over $MgSO_4$. The insoluble material was filtered off through filter paper. The filtrate was concentrated by using rotary evaporator. The crude product was purified by flash column chromatography (silica gel, eluent: chloroform/methanol/acetone/H_2O = 9/4/4/1) to give [1-(3-(dodecylamino) -3-oxopropyl)-[4,4'-bipyridin]-1-ium] [bis((trifluoromethyl)sulfonyl)imide] (2.3 g; 3.4 mmol 21%) as white solid.

^{1}H NMR (400 MHz, $CDCl_3$): δ = 9.00 (d, J = 6.8 Hz, 2H), 8.87 (d, J = 6.6 Hz, 2H), 8.19 (d, J = 6.8 Hz, 2H), 7.66 (d, J = 6.4, 2H), 6.57 (t, J = 2.4 Hz, 1H), 4.94 (t, J = 6.0 Hz, 2H), 3.14-3.08 (m, 4H), 1.24-1.19 (m, 20H), 0.87 (t, J = 6.8 Hz, 3H).

[3-(1′-(3-(Dodecylamino)-3-oxopropyl)-[4,4′-bipyridin]-1,1′-diium-1-yl)propane-1-sulfonate] [bis((trifluoromethyl)sulfonyl)imide] (V^{2+}ZI-12)

1,3-Propanesultone (0.83g; 6.8 mmol) was added to a solution of [1-(3-(dodecylamino) -3-oxopropyl)-[4,4'-bipyridin]-1-ium] [bis((trifluoromethyl)sulfonyl)imide] (2.3 g; 3.4 mmol) in toluene (5 ml) with stirring at room temperature. The mixture was stirred at 130 °C for 48 h. Through recrystallization from toluene and subsequent recrystallization from THF/ethanol mixed solvent, a white solid (1.2 g, 1.5 mmol) was obtained in 44 % yield.

^{1}H NMR (equimolar complexation with $LiTf_2N$, 400 MHz, methanol-d_3): δ = 9.26 (m, 4H), 8.65 (m, 4H), 4.90 (m, 4H), 3.01 (m, 4H), 4.99 (t, J = 6.0 Hz, 2H), 4.94 (t, J = 7.2 Hz, 2H), 3.14-3.08 (m, 4H), 1.25-1.23 (m, 20H), 0.87 (t, J = 6.8 Hz, 3H).

6. ^{1}H-NMR Measurement

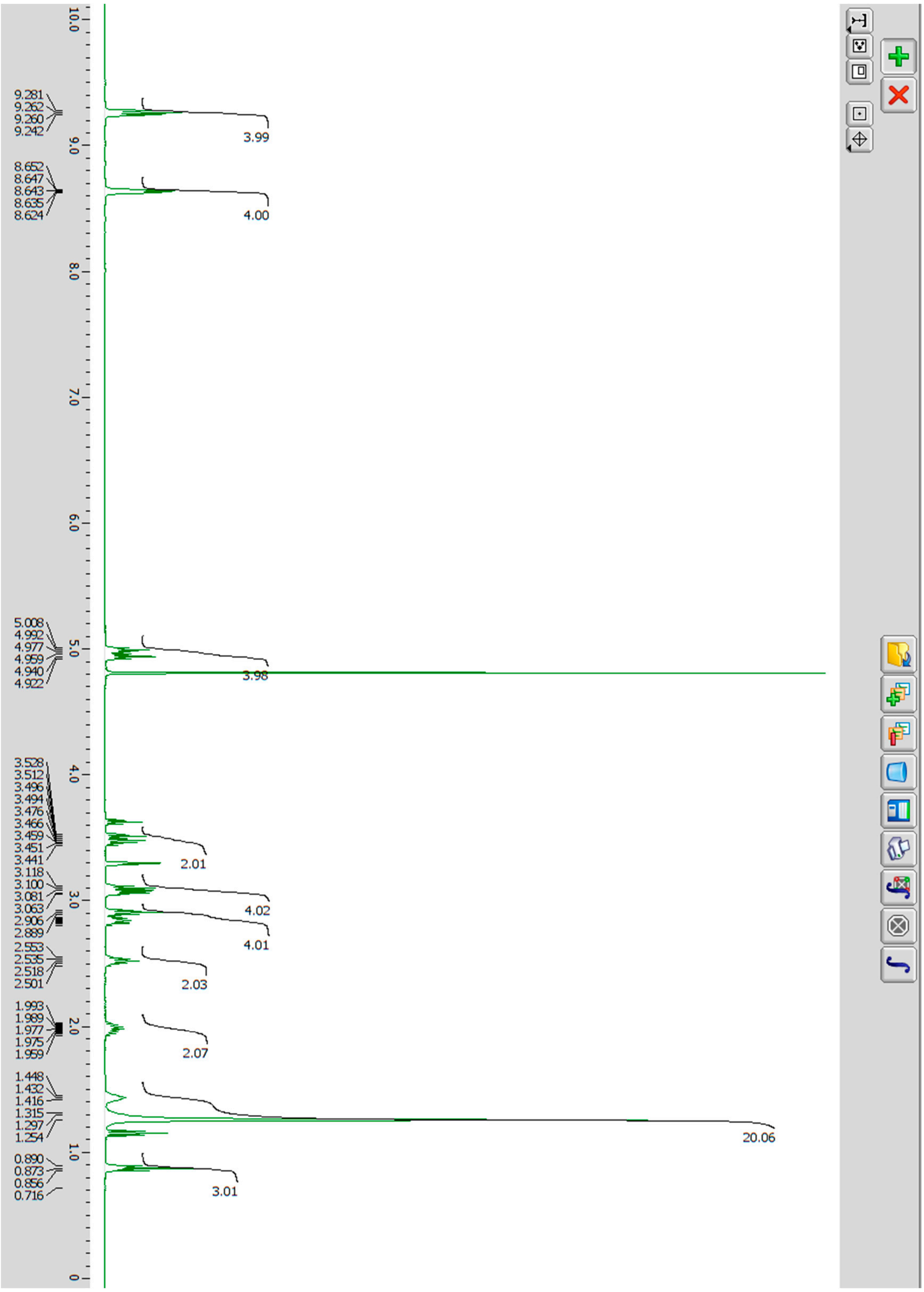

Figure S3. ^{1}H-NMR spectrum of **V^{2+}ZI-12**. An equimolar amount of $LiTf_2N$ is also dissolved in order to increase the solubility of **V^{2+}ZI-12**.

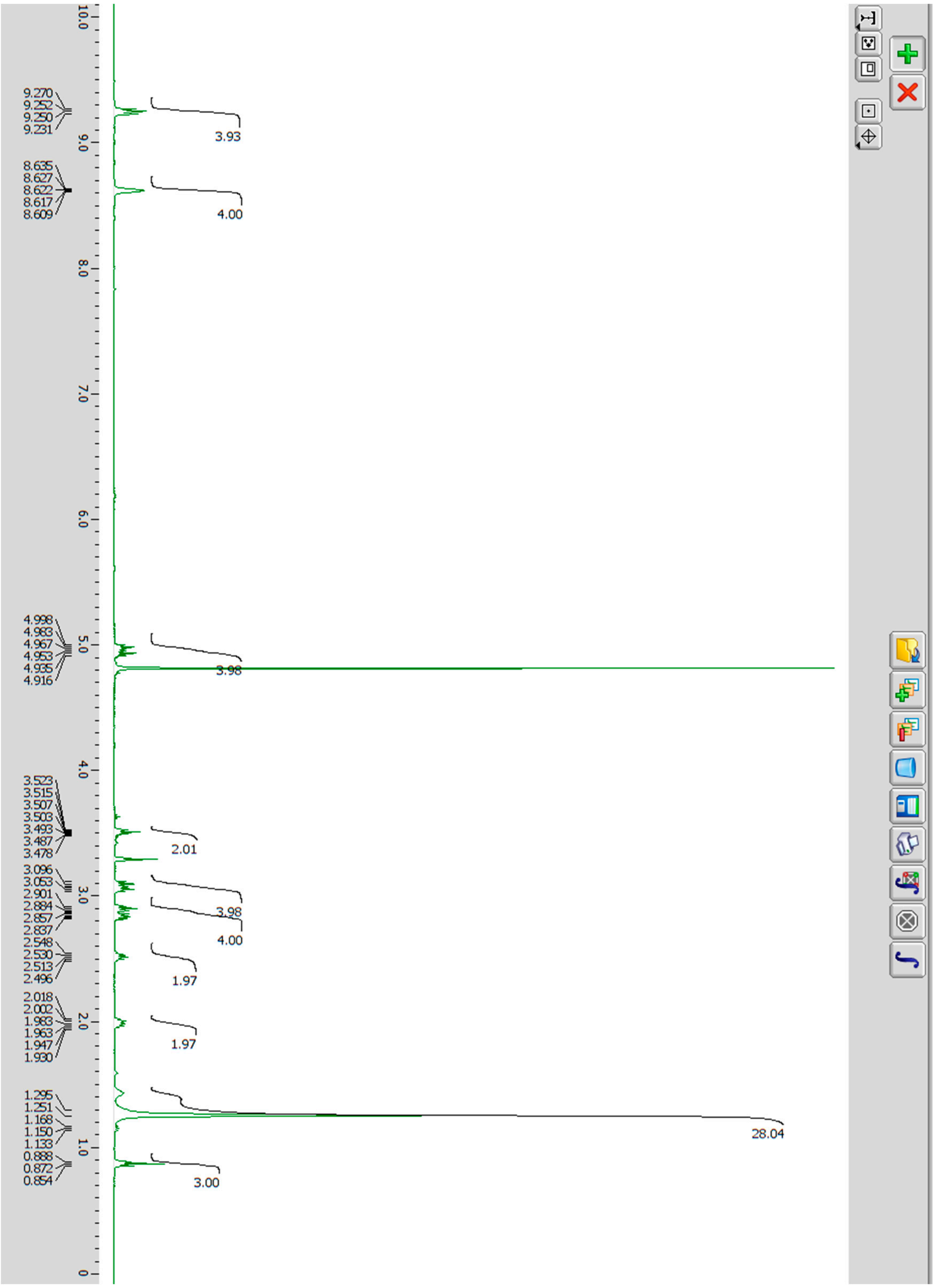

Figure S4. ^{1}H-NMR spectrum of **V²⁺ZI-16**. An equimolar amount of $LiTf_2N$ is also dissolved in order to increase the solubility of **V²⁺ZI-16**.

7. Polarizing Optical Microscope (POM) Observation

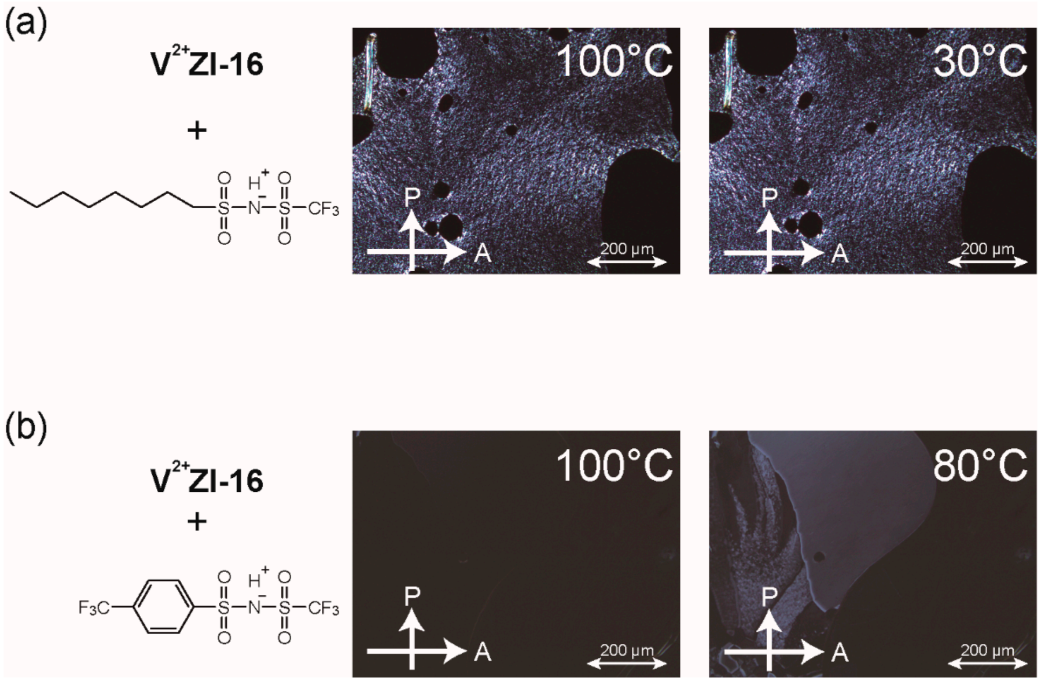

Figure S5. POM images of the mixtures of **V²⁺ZI-16** and various acids; (a) imide-type acids with a long alkyl chain. The mixture shows a Sm phase; (b) imide-type acids with an aromatic ring. The mixture shows a Cub_{bi} phase from 100 to 150 °C and a Col phase from room temperature to 100 °C.

7. *Lyotropic liquid-crystalline behaviors of V^{2+}ZI-16/HTf_2N*

In an aim to confirm the exhibition of Cub_{bi}, lyotropic LC behavior of **V^{2+}ZI-16/HTf_2N** is also examined by using water as solvent. Hydrated **V^{2+}ZI-16/HTf_2N** mixtures (water contents = 5-10 wt%) show Col phases from approximately 30 to 90 °C and Cub_{bi} phase from over 90°C. Interestingly on the cooling process, mosaic-textures were observed. It is a characteristic behavior for the formation of Col phases via cubic phases.

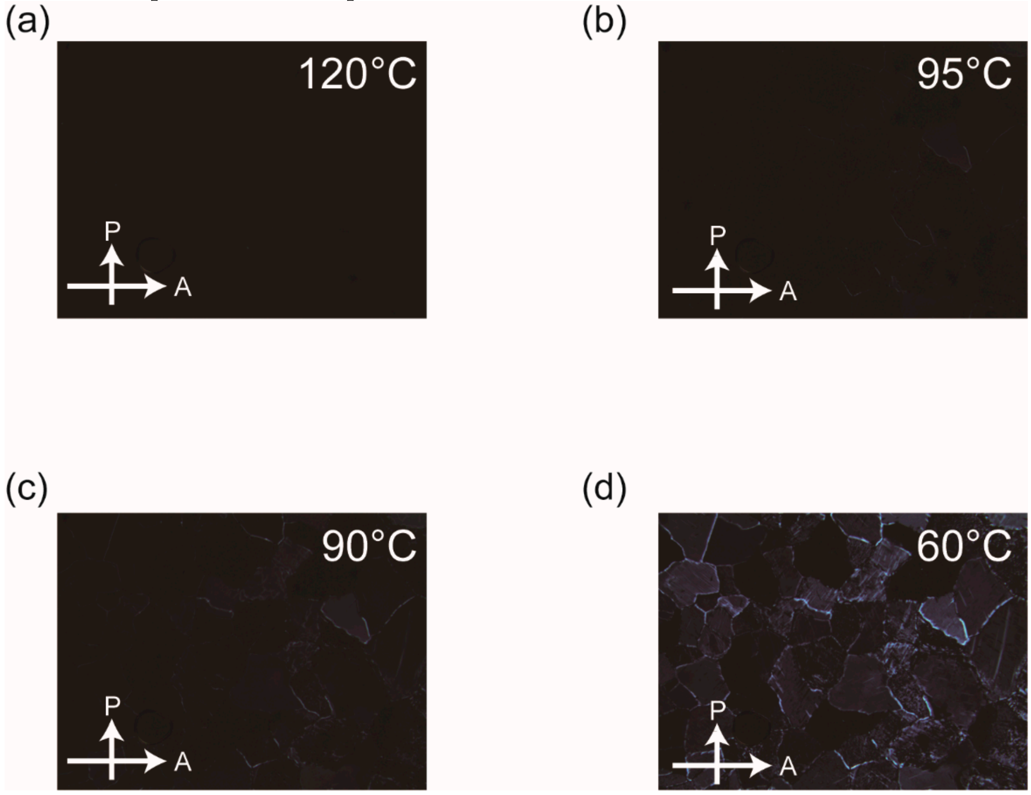

Figure S6. POM images of hydrated **V^{2+}ZI-16/HTf$_2$N** mixtures (water contents are 5-10wt %); (a) at 120 °C in the Cub$_{bi}$ phases; (b) at 95 °C in the Cub$_{bi}$ phases; (c) at 90 °C in the Cub$_{bi}$ and Col phases; (d) at 60 °C in the Col phases.

8. Characterization

8-1. POM measurements for V^{2+}ZI-n and V^{2+}ZI-12/HTf$_2$N.

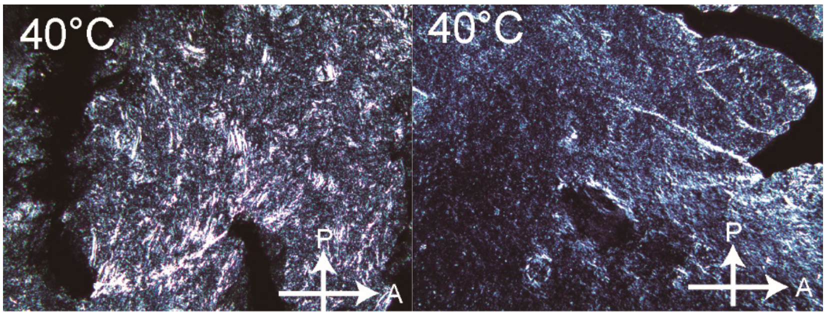

Figure S7. POM images of **V^{2+}ZI-12** (left) and **V^{2+}ZI-16** (right) showing Sm phases at 40°C.

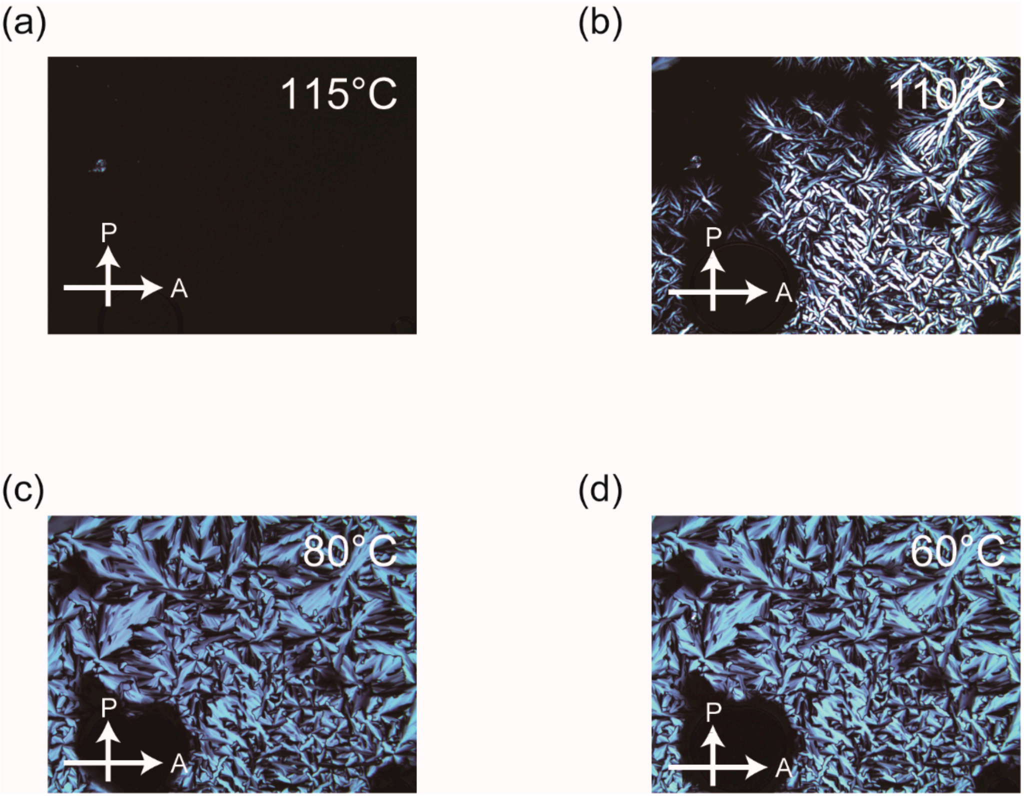

Figure S8. POM images of **V^{2+}ZI-12/HTf$_2$N**; (a) at 115 °C in the Isotropic state; (b) at 110 °C starting in the Col phase; (c) at 80 °C in the Col phases; (d) at 60 °C in the Col phase.

8-2. DSC measurements V^{2+}ZI-n and V^{2+}ZI-n/HTf$_2$N.

Whereas pristine **V^{2+}ZI-*n*** show crystallization (Figure SX), **V^{2+}ZI-*n*/HTf$_2$N** mixtures show glassification at around 0 °C (Figure SX). Generally, liquid-crystalline zwitterions tend to exhibit crystallization owing to strong electrostatic interaction, and also **HTf$_2$N** is crystal at ambient temperature and pressure. However, they exhibit liquid crystalline behavior making homogeneous mixtures. The reason of decreasing crystallinity of two components is that ion-exchange reaction

occurs according to Hard-Soft Acid-Base theory: the viologen dication moiety preferentially forms ion pair with two Tf_2N anions and the sulfonate anion forms ion pairs with proton.

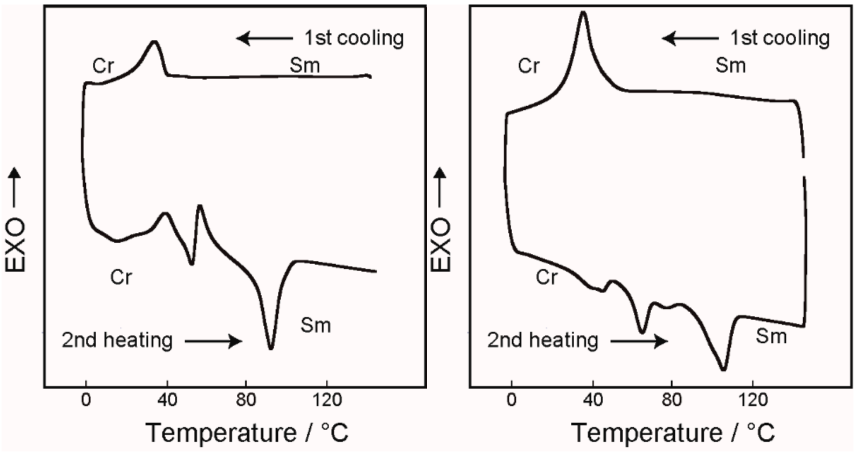

Figure S9. DSC thermograms of; **V[2+]ZI-12** (left) and **V[2+]ZI-16** (right) on heating and cooling.

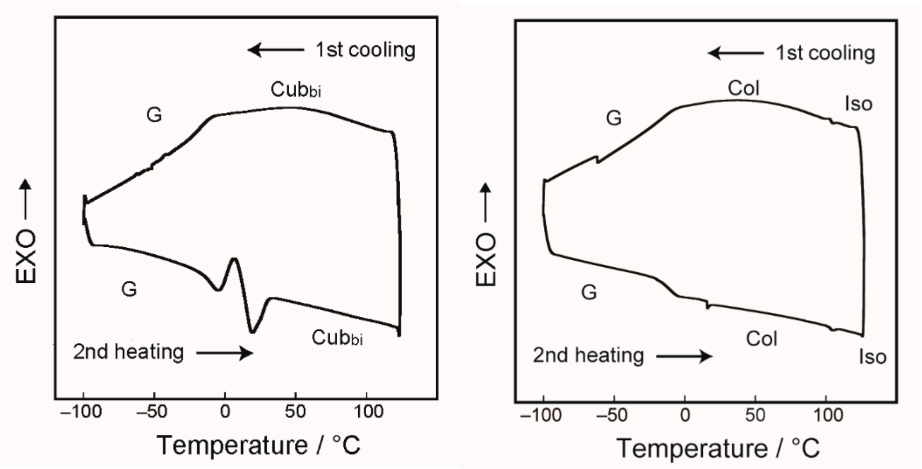

Figure S10. DSC thermograms of; **V[2+]ZI-12/HTf2N** (left) **V[2+]ZI-16/HTf2N** (right) on heating and cooling.

9. LC structures of V[2+]ZI-12/HTf2N mixtures before and after UV irradiation

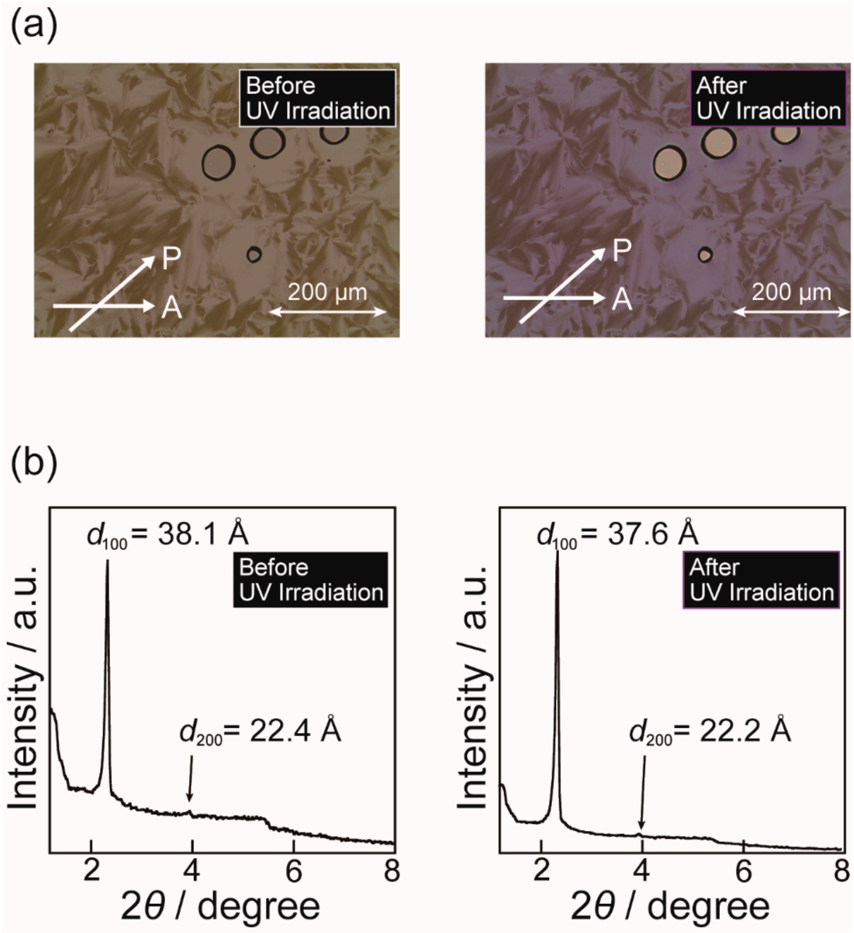

Figure S11. (a) POM pictures before and after UV irradiation. Birefringence formed by each Col domain was maintained while their color turn to purple from colorless. (b) XRD patterns before and after UV irradiation. Two diffraction patterns attributed to (100), (200) of Col plane are observed.

10. Voltage application experiments for V^{2+}ZI-12/HTf$_2$N mixtures

10-1. Experimental way and POM pictures.

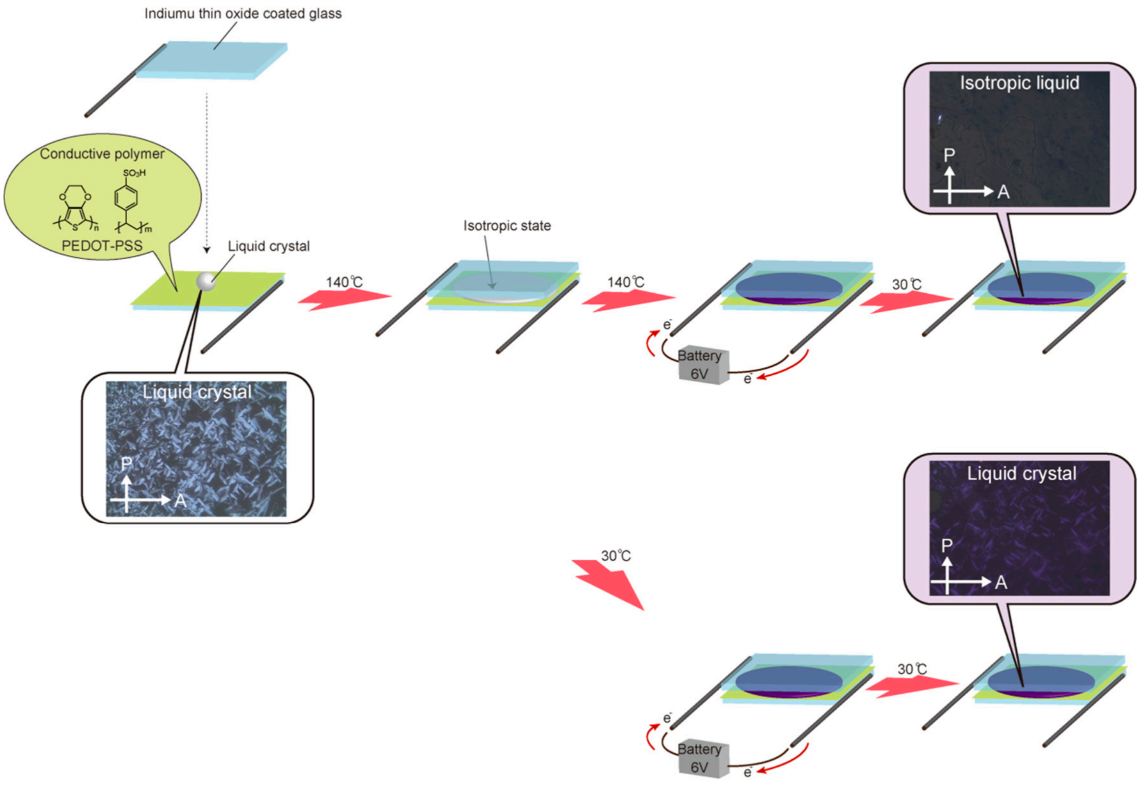

Figure S12. Schematic illustration for voltage application experiments. As applying to voltage, color change immediately occurred to purple from colorless.

10-2. Experimental way and POM pictures.

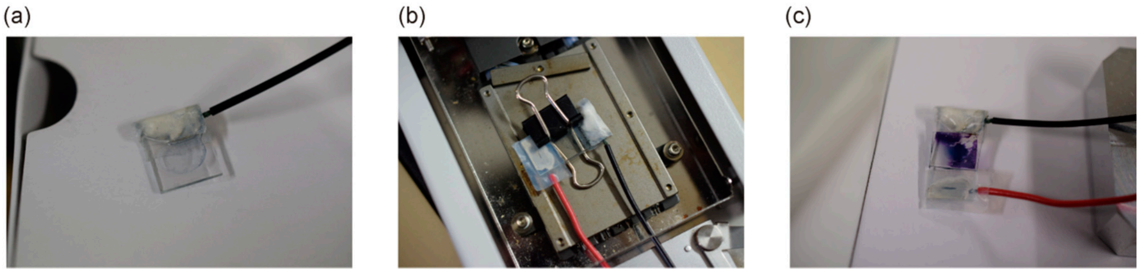

Figure S13. (a) A picture of indium thin oxide glass coated conductive polymer (PEDOT-PSS) electrode. (b) The sample was sandwiched by an indium thin oxide glass and a prepared glass before applying voltage at 140 °C. (c) Color changed sample after applying voltage at 30 °C.

11. Ion conduction measurement for V^{2+}ZI-12/HTf$_2$N mixture

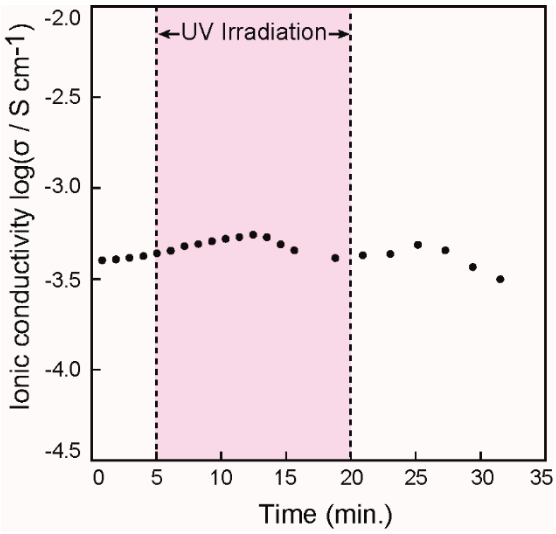

Figure S14. Ionic conductivity of **V^{2+}ZI-12/HTf$_2$N** mixture. UV was irradiated for 5 to 20 min. We checked the color change from colorless to purple accompanying with UV irradiation.

References

[S1-1] Tabushi, I.; Yamamura, K.; Kominami, K.; Electric stimulus-response behavior of liquid-crystalline viologen. *J. Am. Chem. Soc.* **1986**, *108*, 6409–6410, doi:10.1021/ja00280a059.

[S1-2] Casellaa, G.; Causinb, V.; Rastrellib, F.; Saielli, G. Ionic liquid crystals based on viologen dimers: tuning the mesomorphism by varying the conformational freedom of the ionic layer. *Liq. Cryst.* **2016**, *43*, 1161–1173, doi:10.1080/02678292.2016.1161852.

[S1-3] Kijima, M.; Setoh, K.; Shirakawa, H. Synthesis of Novel Ionic Liquid Crystalline Pyrrole Derivatives Having a Viologen Moiety. *Mol. Cryst. And Liq. Cryst.* **2001**, *364*, 911–918, doi:10.1080/10587250108025064.

[S1-4] Tanabe, K.; Yasuda, T.; Yoshio, M.; Kato, T. Viologen-Based Redox-Active Ionic Liquid Crystals Forming Columnar Phases. *Org. Lett.* **2007**, *9*, 4271–4274, doi:10.1021/ol701741e.

[S1-5] Beneduci, A.; Cospito, S.; Deda, M.L.; Veltri, L.; Chidichimo, G. Electrofluorochromism in p-conjugated ionic liquid crystals. *Nat. Commun.* **2014**, *5*, 3105–3157, doi:10.1038/ncomms4105.

[S2-6] Ichikawa, T.; Kato, T.; Ohno, H. 3D Continuous Water Nanosheet as a Gyroid Minimal Surface Formed by Bicontinuous Cubic Liquid-Crystalline Zwitterions. *J. Am. Chem. Soc.* **2012**, *134*, 11354–11357, doi:10.1021/ja304124w.

[S2-7] Matsumoto, T.; Ono, A.; Ichikawa, T.; Kato, T.; Ohno, H. Construction of gyroid-structured matrices through the design of geminized amphiphilic zwitterions and their self-organization. *Chem. Commun.* **2016**, *52*, 12167–12170, doi:10.1039/c6cc06840e.

[S2-8] Ichikawa, T.; Okafuji, A.; Kato, T.; Ohno, H. Induction of an Infinite Periodic Minimal Surface by Endowing An Amphiphilic Zwitterion with Halogen-Bond Ability. *ChemistryOpen* **2016**, *5*, 439–444, doi:10.1002/open.201600054.

[S2-9] Ueda, S.; Kagimoto, J.; Ichikawa, T.; Kato, T.; Ohno, H. Anisotropic Proton-Conductive Materials Formed by the Self-Organization of Phosphonium-Type Zwitterions. *Adv. Mater.* **2011**, *23*, 3071–3074, doi:10.1002/adma.201100942.

© 2017 by the authors. Submitted for possible open access publication under the terms and conditions of the Creative Commons Attribution (CC-BY) license (http://creativecommons.org/licenses/by/4.0/).
